# Significance of additives to enhance the acceptance of poison bait in poultry rodents of Haripur, Khyber Pakhtunkhwa, Pakistan

**DOI:** 10.1371/journal.pone.0272397

**Published:** 2024-01-16

**Authors:** Durr e Shahwar, Syed Ahsan Azeem, Atufa Kawan, Hina Mukhtar, Ahmed Sajawal, Sajida Noureen, Sangam Khalil

**Affiliations:** 1 Department of Forestry and Wildlife Management, University of Haripur, Haripur, KPK, Pakistan; 2 Pakistan Army, Rawalpindi, Pakistan; 3 College of Fisheries, Huazhong Agricultural University, Wuhan, China; 4 College of Food Scienceand Tecnology, Huazhong Agricultural University, Wuhan, China; 5 Depatment of Forestry Range and Wildlife Management, Islamia University Bahawalpur, Bahawalpur, Pakisan; Manonmaniam Sundaranar University, INDIA

## Abstract

Rodent infestation on poultry farms incurs heavy economic losses to this industry by causing feed loss and disease introduction. Development and continuous improvement of rodents control techniques are vital to minimize and control the damages caused by rodents. Here, we test the feed preference of rodents for locally available and palatable food grains viz. millet (whole), wheat (cracked) and rice (broken) and taste additives namely whole egg (5%), eggshell (5%), peanut cracked (5%) and yeast (2%) that were offered mixed in millet-wheat (50:50 by wt.) bait. We tested the preferences of different food additives through a process of feed choice mechanism. We applied two different techniques to compare the preference of mixed feed baits, these techniques included no-choice with multiple choice feeding tests and paired choice with multiple choices feeding tests. The results indicated that consumption of bait with added whole egg was significantly higher (p > 0.05). Further test for its effectiveness as a carrier for rodenticides revealed 56%, 82% and 92%, reduction in rodent activities with zinc phosphide (2%), coumatetralyl (0.0375%) and Brodifacoum (0.005%) respectively. Our results point to a need on continuous improvement of feed baits by using different combinations to effectively control the rodent infestation.

## Introduction

Commercial poultry farming is scaling up rapidly at global level to fulfill the food requirement of the increasing human population. Several farmers in developing countries are taking up poultry to increase their income. The industry is expanding at 2-3% per year and is expected to continue to grow. However, this industry is not immune to the modern world problems and is severely affected by rodent infestations globally [1]. Rodent pests cause heavy losses to poultry farms globally [2, 3]. The precise estimates of these losses caused by rodent pests are not easy to document as they affect at qualitative and quantitative level [4].

Several trials have been conducted to assess the food preferences of rodents to prepare the effective poison baits. The trials in captivity suggested that millet and rice were preferred by *M*. *domesticus*, *R*. *rattus* and *R*. *norvegicus* [5–7]; *M*. *musculus* preferred canaries, seeds, oats and wheat compared to soft wheat, corn, sorghum, sunflower seeds and barns + 5% molasses [5, 8–10] and corn were preferred by *R*. *Exulans* and, *M*. *musculus* [11, 12]. A trial in London found that a mixture of whole wheat, canary seeds, wheat and pinhead, oat meal is preferred by *M*. *domesticus* [10]. The Canary seeds, the wheat, and the rice attracted *M*. *domesticus* in Australia [5]. Whereas in Egypt, *M*. *musculus* chose barley, over, sorghum, seeds, sunflower, and bran with, added 5% molasses [8, 9]. There are limited local case studies to assess the food preference of local rodent species in Pakistan. In one study locally available taste additives namely, broken peanut (5%), yeast (2%), fish meal (5%), egg shell powder (5%), carbon disulfide (CS2) 30ppm and jaggery (5%) were provided to poultry rodents by mixing in millet-wheat (1:1) bait. This study revealed the order of choice of different bait formulations by different rodent species where egg, shell bait was the most preferred whereas the peanut bait was found to be least preferred [13].

Most of the previous studies used taste additives at, 2% (w^t^/wt) to, a bait, base composed, of, equivalent, amounts, of, broken, rice, and wheat, flour. Taste additive1such as eggs (3%) are known to make bait more palatable to commensal, (*R*. *rattus*) and field rodent (*N*. *indica* and, *B*. *bengalensis*) [14]. In another study in, India 2% egg albumin and egg shell, powder in cereal bait enhanced the acceptance and efficacy of 2% zinc phosphide against *R*. *rattus* trapped in poultry farm conditions [15]. In Pakistan, 5% egg, shell, grain bait was evaluated for its efficacy as a carrier for zinc phosphide (2%) and coumatetralyl rodenticides (0.0375%). The decline in rodent activity after zinc phosphide and coumatetralyl, treatment was 70% and 82% respectively, [13].

However, prolonged use of same bait combination at a particular location decreases the efficacy of that bait leading to an increased rodent infestation. To keep baits attractive and affective for rodent control a continuous research process should be carried out to develop multiple poison bait combination for effective rodent control [16]. The major objective of this, study, was to establish preferences among millet, wheat, and rice Furthermore, we tested the additions of some substance such as whole egg, eggshell, yeast, and peanut contribute to increase the consumption of these grains, and whether these additives promote the consumption of rodenticide baits.

## Materials and methods

Advanced Studies Research Board ethical committee of University of Haripur, KPK, Pakistan approved all experiments. All experiments were performed in accordance with relevant guidelines and regulations.

We conducted this study in the poultry farms of Haripur in the year 2018 and 2019. The study area covered 3465.6 km^2^ (33^0^36’N, 73^0^04’E). Most of the poultry farms were located in areas surrounded by harvested wheat, corn, sorghum and groundnut crops.

**Fig 1:**
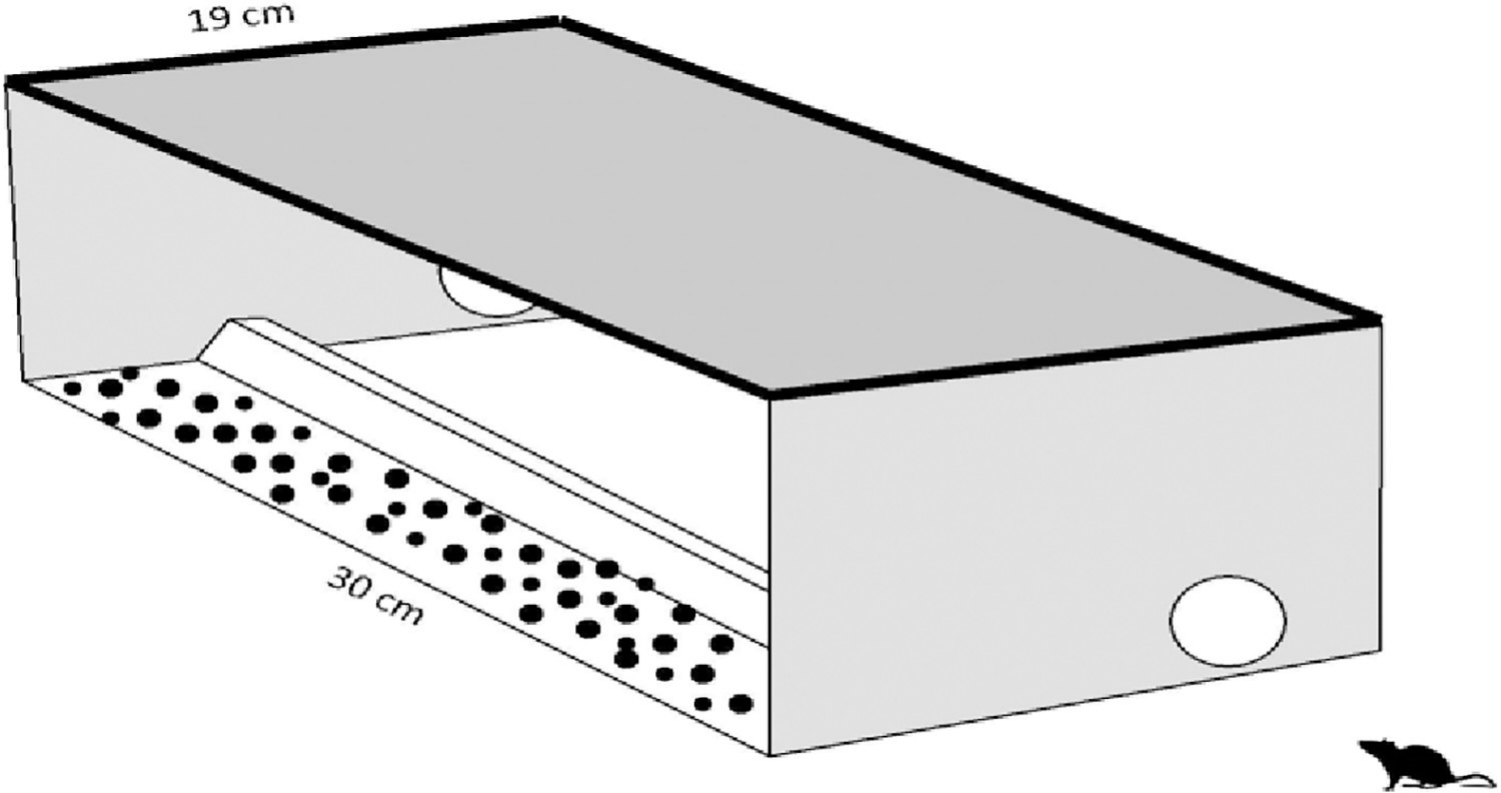
Design of bait box used for rodents on poultry farms of Haripur, KPK, Pakistan.

The design of bait stations plays an important role in bait consumption of commensal rodents. Our locally made wooden box bait stations are 30 cm long 19 cm wide and 9.5 cm high with two entrances (5 cm in diameter) at opposite ends of the box [13]. There was an internal 6 cm long bait positioning channel and a lockable lid at the bait station (Fig 1). To determine the spillage and keep the baits out of the approach of the chickens, the grains were offered in bait boxes [17] providing bait inside bait boxes is an efficient form of rodent management [18].

We selected three types of locally available grains that among the most palatable to rodents [13, 19–21] to further assess their palatability and acceptance by the rodent populations in poultry farms in our study area. We tested wheat (*Triticum aestivum*) in cracked form, rice (*Oryzasativa*) in broken form and whole millet seed (*Pennisetum typhoideum*) in no-choice and multiple-choice feeding tests to determine the most preferred grain.

### No choice feeding

We offered three grains viz. millet, wheat and rice. Wheat grains were ground/cracked to a particle size similar to that of millet grain, while rice (in 1broken form). All three grains were cleaned of debris and dust before use. In no-choice test at each bait station each grain was offered in one bait box for 17 days at the three selected poultry farms. Bait boxes were placed at appropriate where there was evidence of movement/activity of rodents. Initially, we offered 100 g at each bait station, but the amount was subsequently adjusted to ensure a surplus amount of feed for the rodents. Bait materials were replenished twice mean while consumption and spillage were measured by using electronic digital balance. On each visit position of bait boxes were changed to avoid the place preference [13, 22]. The grain consumption was estimated by taking the difference in amount of bait placed and the amount of bait left over in each bait station on the day of observation. Numbers of bait stations varied in accordance with the size of the poultry farms and apparent signs of the rodent activities.

There were eight bait stations at the first farm/replicate, six at second and third farm/replicate. The bait stations were located inside the poultry farm buildings (feed store and poultry shed) and even at open areas outside the buildings, if 1necessary. However, it was preferred to place the bait boxes inside the buildings to avoid chances of theft, exposure to non-target animals and extreme weather conditions.

### Multiple choice feeding test

Multiple-choice feeding studies were conducted on the same poultry farms approximately four weeks after the no-choice trials. Millet and wheat were delivered simultaneously in sets of two bait boxes located at the appropriate locations at the three poultry farms in this feed test. We used eight bait stations at farms one and two while six at the third in pairs of four and three, all of the bait boxes were placed in pairs.

### Additives

We picked four locally accessible bait additives which have proven to have some efficacy, i.e., egg shell powder, peanut, and whole egg [13, 23] and yeast [13, 24]. These additives were used at 2% and 5% concentrations to test their potential. Based on the no-choice experiment and multiple-choice feeding test results, millet (whole) and wheat (cracked) were used as bait.

### Paired choice feeding test

We included four poultry farms to evaluate a suitable bait additive for attraction and enhancement of bait intake. All baits were prepared by adding and mixing thoroughly a measured quantity (2% or 5% w/w) of the additives to the bait. The feeding tests were performed simultaneously at four selected poultry farms using a paired choice procedure offering plain bait (mix of millet and wheat) in one box and additive bait in the second. We placed bait boxes at locations where there were signs of activity of the rodents. The number of bait stations ranged from six to eight.

### Multiple-choice feeding tests

This experiment was performed at three poultry farms to further refine the results of paired-choice feeding tests. A mix of millet and wheat with additives were offered simultaneously in separate bait boxes placed together on one bait station. There were four sets of bait stations at each poultry farm to place five bait boxes per station.

### Rodenticides

The practical field efficacy of the most palatable candidate bait ingredients was tested by incorporating commonly used rodenticides. Acute rodenticide (zinc phosphide) and anticoagulant (coumatetralyl 0.0375% and brodifacoum) rodenticide.

### Zinc phosphide preparation

We used millet and wheat (50:50) mixed with 5% whole egg as the carrier for zinc phosphide. We prepared a 5 kg batch of bait comprising 45.5% millet, 45.5% cracked wheat, 5% egg shell, 2% vegetable oil1 and 2% zinc phosphide. 80% pure, marketed as ‘Agzinphos’, A. G. Services.

### Efficacy of zinc phosphide

Zinc phosphide bait was experimented with at three poultry farms using pre-baiting for six days to mitigate neophobia and increase the chance of rodents consuming a lethal dose [13, 25]. Zinc phosphide bait was then offered for three consecutive days in eight bait boxes at farm one, seven at farm two and ten bait stations at farm three. To assess pre-treatment rodent activities: we placed vinyl tiles (15 x 15 cm) with chalk powder spread on their surfaces in feed/egg stores and poultry sheds for three consecutive nights. We observed these tiles every morning for rodent footprints. Post-treatment rodent activity was recorded immediately after the removal of the bait boxes. Change in ratio used to estimate the success of the rodenticide bait.

### Coumtetralyl/raccumin (0.0375%) preparation

A 5 kilograms batch of bait containing 0.0375% coumatetralyl was prepared by mixing 46.5% millet (whole), 46.5% wheat (cracked), 5% whole egg, 2% vegetable oil and 0.0375% coumatetralyl /Racumin, as 0.75% powder concentrate; Bayer, Germany).

### Efficacy of coumtetralyl/raccumin (0.0375%)

An additional set of three poultry farms was selected for evaluation of bait containing 0.0375% coumatetraly using 6 bait boxes at the first farm, 8 at second farm and 11 at third farm. Pre-treatment rodent activity was recorded using tracking tiles as before. The bait was placed for 11 consecutive days, and then tracking tiles were placed again for three nights.

### Brodifacoum (0.005%) preparation

A 5 kiolgram batch of bait containing 0.005% brodifacoum was prepared by mixing 46.5% millet (whole), 46.5%) wheat (cracked), 5% whole egg, 2% vegetable oil and 0.005% brodifacoum.

### Efficacy of brodifacoum (0.005%)

For the assessment of bait containing 0.005% brodifacoum, another set of three poultry farms were selected using 8 bait boxes at the first farm, 10 at the second farm and 7 at the third farm. Using tracking tiles as before, pre-treatment rodent activity was recorded. For 11consecutive days, the baits were placed and afterward tracking tiles were set again for three nights.

### Statistical analysis

- We employed ANOVA to assess the effect of farms, days of feeding and grain type on bait consumption for no choice and multiple choice feeding tests of plain baits and multiple choice feeding tests of additives. To analyze non independent data, because the feeding tests were conducted in the same farms, it was conducted as repeated measure analysis.
- The t test was used to compare the mean consumption of bait with additives versus plain bait in the paired choice test.
- Rodent activity on tracking tiles before and after treatment was compared using paired, two tailed t tests. Changes in rodent activity indices were assessed using the method of [13, 26].

## Results

### No-choice test

Examination of the intake of three palatable grains (millet in whole form and wheat and rice in cracked forms) over the 2.5 weeks (17 days) period revealed that farms, days of feeding and grain types all had a significant effect on bait take (Tables 1 & 2: F = 7.414, df = 2, P = 0.004). Bait consumption at farm 3 was higher than at farms 1 and 2. Rodents ate significantly more millet and wheat than rice (Table 3).

**Table 1:** Utilization (g) of three1 grain forms measured in the no-choice^1^ feeding test at three1 poultry1 farms of Haripur, Pakistan.

Days1 of the Feeding1	Millet1 (Mean ± SE)	Positive^1^ Boxes with bait n (%)	Wheat1 (Mean ± SE)	Positive1 Boxes with bait n (%)	Rice1 (Mean ± SE)	Positive^1^ Boxes with bait n (%)
1	0	0	0	0	0	0
2	0	0	0	0	0	0
3	0	0	0	0	0	0
5	814(90.44 ± 18.72)	9(45)	780(130 ± 10.80)	6(30)	774(154 ± 9.61)	5(25)
7	3175(397 ± 18.12)	8(40)	1292(258 ± 12.94)	5(20)	1050(150 ± 13.36)	7(35)
9	4237(282 ± 18.23)	15(75)	3565(324 ± 19.16)	11(55)	1148(127 ± 9.93)	9(45)
11	6142(361 ± 18.86)	17(85)	3069(236 ± 13.04)	13(65)	2088(190 ± 13.75)	11(55)
13	4355(311 ± 17.60)	14(70)	3067(340 ± 10.34)	9(45)	2030(254 ± 15.52)	8(40)
15	6616(551 ± 18.10)	12(60)	4940(617 ± 14.25)	8(40)	1535(128 ± 13.87)	12(60)
17	6972(387 ± 18.28)	18(90)	3169(226 ± 15.56)	14(70)	1754(135 ± 15.49)	13(65)
Total	32311g		19882g		10379g	

**Table 2:** Analysis of variance of bait takes showing variation due to grain type, replicate/farm and days of feeding.

Source of variation	Sum of square	Degree of freedom	Mean square	Variance ratio (F)	Probability (P)
Replicates/farms	3.2972E7	2	1.546E7	51.904	<0.001
Grains type	50,35,204.143	2	13,24,232.521	7.414	0.004
Days of feeding	3.093E7	9	17,21,141.023	4.112	<0.001
Days*grains type	1.132E7	18	211,241.132	0.434	0.433
Replicates/farms*days	1.140E7	18	551,313.310	2.187	<0.001
Error	1.133E7	70	111,100.443		
Total	2.034E8	99			

**Table 3:** Scoring of the use of grain baits (in no choice feeding test) and relative variation between rodent pest intakes at poultry farms of Haripur, Pakistan.

Grains	No. of Observations	Least Significant Difference test		
		Subset for alpha = 0.0511		
11	12	13
Millet	110	3231.1^a^		
Wheat	110		1988.2^b^	
Rice	110			1037.9^bc^

Neophobia (avoiding a new thing/object in the environment) is often displayed by rodent species and explains the low consumption of grain during the initial four days of millet presentation in the no choice test. The first signs of rodent visits to bait boxes were recorded on day 5 of baiting, whereas more than 65% of the bait boxes showed rodent activity on day 11 and 70% by day 17, with a similar level of activity for the remainder of the trial. It was reflected in a gradual but significant increase in millet consumption with time, and we observed a similar pattern in the consumption of the other two grains (wheat and rice). The difference in consumption of the three grains during the study was presumably influenced by taste, odour and palatability of the individual grain type and differences between farms and availability of other food types.

### Multiple-choice feeding test

The results of the multiple choice test confirmed those of the no choice tests, with millet again the most preferred grain to poultry rats (Tables 4 & 5: F1 = 6.702, df = 1, p = 0.024). Days of feeding and grain types significantly influenced bait consumption. The consumption of millet and wheat did not differ significantly.

**Table 4:** Utilization (g) of two1 grain forms measured under 1multiple-choice^1^1 feeding1 testat three11 poultry1 farms1of Haripur, Pakistan.

Days11 of the feeding^1^11	Millet11 (Mean ± SE)	Positive1^1^ Boxes with bait n (%)	Wheat11 (Mean ± SE)	Positive11 Boxes with bait n (%)
5	725(60 ± 17.85)	12(60)	485(48 ± 14.61)	10(50)
7	965(64 ± 14.57)	15(75)	590(49 ± 14.21)	12(60)
9	913(65 ± 16.01)	14(70)	280(31 ± 13.69)	9(45)
11	5251(477 ± 14.87)	11(55)	921(115 ± 11.87)	8(40)
13	4170321 ± 15.74)	13(65)	1022(93 ± 12.50)	11(55)
15	5111(301 ± 17.25)	17(85)	1270(91 ± 13.81)	14(70)
17	3231(215 ± 16.52)	15(75)	1586(122 ± 14.74)	13(65)
Total	20366g^A^		6154g^AB^	

Same letters (alphabets) are non-significant to each other.

**Table 5:** Analysis of variance of bait takes showing variation due to grain type, replicate/farm and days of feeding.

Source of variation	Sum of square	Degree of freedom	Mean square	Variance ratio (F)	Probability (P)
Replicates/farms	5,033.224	2	11,214.322	0.310	0.635
Grains type	532,322.513	1	123,132.132	6.702	0.024
Days of feeding	324,242.502	9	115,830.377	1.431	0.015
Days*grains type	142,210.463	9	13,323.333	0.221	0.860
Replicates/farms*days	204,043.032	18	12,431.342	0.330	0.133
Error	12,63,470.440	53	32,325.113		
Total	42,62,403.550	81			

These tests were performed on the same farms as the no choice tests, and after a 2-3 weeks interval rodents did not show 1initial neophobia towards the bait stations, with 60% of bait stations showing rodent activity on day 5 of the trial. Whereas in the no choice trial the amount of each of the grains eaten showed a gradual increase in food consumption.

### Paired choice feeding test

The results of the paired-choice feed tests (bait combined with additive versus bait without additive) are listed in the following subsections.

### Whole egg

Comparison of total grain ingestions showed that the ingestion of whole egg bait was significantly higher than that of plain bait (Table 6; t =-4.1, df = 6, p = 0.001). After some initial hesitation, both bait boxes showed signs of rodent activity, although a marked preference for the additive bait over the plain bait was recorded, consumption of plain bait fluctuated while that of additive bait increased linearly (Table 6). The mean consumption of the bait with egg additive (18.47 ± 1.85g) was higher than that of plain bait (8.2 ± 1.91 g).

**Table 6:** Utilization (g) of plain1 bait1vs. bait mixed1 with 1additive (whole 1egg) measured under 1paired-choice1 feeding test at poultry1 farms of Haripur, Pakistan.

Days1 of the feeding	Plain bait (Mean ± SE)	Positive Boxes with bait n (%)	Whole egg 15% (Mean ± SE)	Positive Boxes with bait n (%)
5	105 (13 ± 19.98)	8 (80)	51 (6 ± 15.22)	9 (90)
7	107 (12 ± 18.72)	9 (90)	54 (5 ± 16.50)	10 (100)
9	86 (12 ± 19.54)	7 (70)	353 (44 ± 12.22)	8 (80)
11	201 (25 ± 21.33)	8 (80)	274 (27 ± 14.56)	10 (100)
13	76 (8 ± 18.07)	10 (100)	467 (67 ± 11.90)	7 (70)
15	391 (43 ± 16.20)	9 (90)	695 (77 ± 11.72)	9 (90)
17	271 (27 ± 16.21)	10 (100)	881 (88 ± 14.37)	10 (100)
Total	1237g (8.2 ± 1.91)		2775g (18.47 ± 1.85)	

### Yeast

Cumulative consumption of bait added with 2% yeast powder was significantly higher than that of the plain bait (Table 7: t = -13.823, df = 6, p = 0.009).

**Table 7:** Utilization (g) of plain1 bait1vs. bait1 mixed with 1additive (yeast) measured under1 paired-choice1 feeding test at poultry1 farms of Haripur, Pakistan.

Days1 of the feeding1	Plain bait (Mean ± SE)	Positive Boxes with bait n (%)	Yeast 12% (Mean ± SE)	Positive Boxes with bait n (%)
5	56 (7 ± 14.33)	8 (80)	70 (8 ± 11.11)	9 (90)
7	78 (9 ± 14.95)	9 (90)	88 (9 ± 11.83)	10 (100)
9	67 (9 ± 16.43)	7 (70)	85 (10 ± 11.31)	8 (80)
11	91 (11 ± 11.44)	8 (80)	99 (10 ± 13.75)	10 (100)
13	150 (15 ± 12.20)	10 (100)	87 (9 ± 12.25)	10 (100)
15	130 (14 ± 13.69)	9 (90)	170 (19 ± 12.74)	9 (90)
17	72 (7 ± 13.17)	10 (100)	195 (28 ± 14.67)	7 (70)
Total	644g (4.2 ± 1.68)		794g (5.47 ± 1.30)	

### Peanuts

Bait comprising mixed millet and wheat with 5% cracked peanuts added was eaten in large quantity than the plain bait mixture (Table 8; t = - 4.05, df = 6, p= 0.007). On the 3^rd^ day all 5 bait boxes (for plain & additive baits) showed signs of rodent activity. Consumption of both bait forms fluctuated with no consistent trend over time (Table 8).

**Table 8:** Utilization (g) of plain1 bait1vs. bait1 mixed with ^1^additive (peanut) measured 1under ^1^paired-choice1 feeding^1^ test at poultry1 farms of Haripur, Pakistan.

Days1 of the feeding1	Plain bait (Mean ± SE)	Positive Boxes with bait n (%)	Peanut 5% (Mean ± SE)	Positive Boxes with bait n (%)
5	49 (5 ± 13.41)	9(90)	69(8 ± 11.97)	
7	89 (10 ± 12.77)	9(90)	99 (10 ± 13.63)	9 (90)
9	73(7 ± 14.03)	10(100)	103 (10 ± I 3.78)	10 (100)
11	74(7 ±I I .3 I)	10(100)	85 (8 ± 12.47)	10 (100)
13	61 (6 ± I 2.57)	10(100)	7 I (7 ± 10.60)	10 (100)
15	90 (10 ± 13.22)	9(90)	1 60 (1 6 ±+ I I .41 )	10 (100)
17	110 (9 ± 10.55)	10(100)	1 15 (1 I ± I 2.79)	10 (100)
Total	546g (3.17 ± 1.22)		702g (4.1 I ± 1.18)	

### Egg shell

A comparison of the total grain intake showed that bait consumption was significantly higher with added egg shells than the plain bait (Table 9: t = -1.95, df = 6, p =0.007). All the bait boxes showed signs of rodent activity after some initial hesitation, although a marked preference was observed for the additive bait over the plain bait (Table 9). The plain bait consumption fluctuated, while additive bait consumption rose linearly. The mean consumption of the bait with egg additive (16.05 ± 0.82 g) was higher than the plain bait (11.47 ± 1.39 g).

**Table 9:** Utilization (g) of plain1 bait1 vs. bait1 mixed1 with additive1 (egg1 shell) measured under1 paired-choice1 feeding test1 at poultry^1^ farms of Haripur, Pakistan.

Days1 of the feeding1	Plain1 bait (Mean ± SE)	Positive Boxes with bait n (%)	Egg Shell 15% (Mean ± SE)	Positive Boxes with bait n (%)
5	129 (32 ± 13.76)	4 (80)	149 (29 ± 12.04)	5 (100)
7	114 (29 ± 12.81)	5 (100)	140 (28 ± 13.50)	5 (100)
9	110 (22 ± 11.25)	5 (100)	134 (34 ± 11.93)	4 (80)
11	170 (34 ± 12.94)	5 (100)	190 (38 ± 11.81)	5 (100)
13	105 (21 ± 11.93)	5 (100)	220 (44 ± 10.83)	5 (100)
15	120 (30 ± 11.08)	4 (80)	230 (46 ± 12.44)	5 (100)
17	135 (27 ± 9.61)	5 (100)	270 (54 ± 11.51)	5 (100)
Total	883g (11.47 ± 1.39)		1333g ( 16.05 ± 0.82)	

### Multiple choice feeding test

When all baits with additives and plain bait were tested together consumption of bait with 5% whole egg was the highest (Table 10); there was also significant differences between the different baits (F = 37.774, df = 4, p < 0.001). Farms, days of feeding and additive types, had a significant influence on bait take (Table 11).

**Table 10:** Utilization (g) of different bait1^1^formulations, i.e. simple bait1 versus bait combined with four additive types (whole egg, 1egg shell, yeast and 1peanut) under1 multiple-choice^1^ feeding experiments at poultry1 farms of Haripur, Pakistan. Total Bait Intake (Mean ± SE); Total^1^ Number of Bait1 Stations = 115.

Days1 of the Feeding	Whole egg1	Egg shell1	Yeast^1^	Peanut1	Plain bait
1	0.00	0.00	0.00	0.00	0.00
2	1281 (260±15)	512 (170±12)	201 (100±13)	3501	440 (150±14)
3	12660 (840±15)	6781 (520±110)	3751 (540±18)	2751 (310±15)	2910 (220±13)
5	15441 (1030±117)	4960 (380±111)	3620 (400±18)	3030 (340±17)	2391 (170±13)
7	15850 (1060±120)	9631 (740±116)	4921 (550±114)	5681 (630±16)	2101 (140±11)
9	17521 (1170±125)	5311 (410±18)	5680 (630±16)	4351 (480±110)	2841 (190±13)
11	17440 (1160±131)	9430 (730±114)	4241 (430±112)	4171 (460±110)	6471 (430±17)
13	17331 (1160±118)	9681 (750±120)	4801 (530±110)	2370 (260±15)	3490 (230±15)
15	19110(1270±125)	11850 (910±126)	2921 (320±16)	5151 (570±116)	4191 (280±14)
17	15501 (1030±114)	12251 (940±135)	3710 (410±111)	3931 (440±111)	2741 (180±13)
Total	132130 (31.15 ±113.4)*	70401 (16.8 0±110)*	32030 (7.60±15.9)*	31781 (7.61±15.6)*	27572 (6.61 ±13.5)*

Number of positive bait boxes for whole egg = [50-115], egg shell [30-113], yeast [2,7 &09], peanut [10–19], plain bait [3, 13, 14 & 15]

**Table 11:** Scoring of the use of plain1 and additive1 mixed bait1 (in the multiple1 choice feeding1 test) and relative1 variations between rodent pest intakes at poultry farms of Haripur, Pakistan .

Additives	No. of Observations	Duncan’s multiple range test		
		Subset for alpha = 0.0511		
11	12	13
Plain bait	110	1275.70 ^C^		
Peanut	110	1317.80 ^C^		
Yeast	110	1320.30 ^C^		
Egg shell	110		1704.00 ^B^	
Whole egg	110			11321.30 ^A^

Comparison of bait types using Duncan’s Multiple Range test suggested the following order of preference; whole egg bait > egg shell bait > yeast bait > peanut bait > plain bait. Consumption of whole egg bait was significantly higher than egg shell bait and consumption of both of these formulations was significantly higher than the other three bait formulations. All 15 bait boxes containing egg shell and yeast baits showed positive signs of rodent activity within three days of their placement (Table 10) while with plain bait it took 10 days. The rest of the bait types showed varying attractiveness to the rodents.

### Efficacy of the most palatable bait with 2% zinc phosphide

Unlike the previous feeding1tests the rodent populations1on1the three poultry1farms1visited the bait boxes on the first day1of their1placement. Consumption of plain^1^bait (Table 12) 1during the first three days of pre-baiting increased from 33 g per bait box per night,, 41 g per bait box per night on day 4. When the rodenticide1bait was offered on the 7th day of the test there1 was a reduction in bait1consumption to 20±13.22g/night/bait box. Afterwards there^1^ was a 1gradual1decrease in consumption of poison1bait presumably due to the 1rodenticide-related mortality of rodents. When the experiment reached the 8th and 9th night, there was negligible consumption of poison bait.

**Table 12:** Utilization of bait without poison and 12% zinc, phosphide bait (millet-wheat 11:1^0^ with 15% whole egg) at three^1^ selected poultry farms of Haripur, Pakistan.

Baiting1 phase	Days1 of the feeding	Total bait1 boxes1 (n)	Positive1 boxes1 with bait (n%)	Total1 consumption1 in g (Mean ± SE)	Dead1bodies of rodent1 species recovered (n)		
					*M*.*m*	*R*.*r*	*R*.*n*
Pre baiting1	1	25	23(92)	762(33 ± 9.39)			
	2	25	25(100)	880(35 ±10.92)			
	3	25	25(100)	995(40 ± 10.8)			
	4	25	25(100)	1020(41 ± 10.98)			
	5	25	25(100)	765(31 ± 9.74)			
	6	25	24(96)	980(31 ± 11.39)			
Total(Mean ± SE)				5402(35 ± 0.78)			
Zn_2_P_3_ baiting	7	25	20(80)	396(20 ± 13.22)	9	3	4
	8	25	24(96)	576(24 ± 11.74)	7	5	1
	9	25	24(96)	378(16 ± 12.12)	5	4	0
Total(Mean ± SE)				1350(20 ± 0.76)	21	12	5

* Values1 given in mean1 consumption1 (g) per day^1^ per 1positive bait1 box

** R. r. = Rattussrrattus; M.m. = Mussmmusculus; R.n. = Rattussnnorvegicus

The pre-treatment and post-treatment rodent monitoring showed a significant reduction (t = 12.8, df = 2, P = 0.006) in rodent activity in the post-treatment period compared to the pre-treatment period. The overall rodent activity reduction could be due to 2% zinc (Table 13) phosphide (mixed with wheat-millet & whole egg) treatment was estimated at 56%.

**Table 13:** Data on 1pre-treatment1 and 1post-treatment1tracking1tiles1 that indicate a decrease in rodent activity due to 12% zinc11pphosphide mixed1 in bait1 base (millet-wheat 11:10 with 15% whole egg) at three1 selected poultry1 farms of Haripur, Pakistan.

Days1 of observation	Tracking1 tiles^1^			
	Total	Pre-treatment	Post-treatment	Decrease (%)
		n (index)	n (index)	
1	45	39(0.86)	21(0.46)	
2	45	36(0.79)	16(0.35)	
3	45	38(0.85)	12(0.49)	
Total(Mean ± SE)		113(38 ± 1)	49(16 ± 2)	56

Decrease in rodent1 activity1 evaluated following [26].

### Efficacy of the most palatable bait as carrier of 0.0375% coumatetralyl

Mean daily consumption of coumatetralyl bait for the first eleven days was 22 ± 3 g/positive bait 1box/night (Table 14). Afterwards, the intake of coumatetralyl bait started to decrease, and only 112 g ± 1 g/ positive bait box/night got consumed on the 14th night of treatment.

**Table 14:** Utilization of poison bait 10.03751% coumatetralyll (millet-wheat 11:10 with 15% egg1 shell) during^1^ poison1 baiting1 nights1 at three11replicates/poultry1 farms of Haripur area.

Poison1 baiting1	Days1 of the feeding	1Total bait1 boxes1 placed1	Positive boxes1with bait1(n %)	Total1 consumption1 g (mean ± SE)	Dead bodies of rodent species1 recovered (n)		
					*Mus*. *M*	*R*. *r*	*R*. *n*
Coumatetralyll	14	135	24 (69)	1308 (130 ± 13)	40		
	15	135	32 (94)	1466 (150 ± 13)	30	20	10
	16	135	31 (89)	1488 (160 ± 14)	30	30	30
	17	135	34 (97)	1721 (210 ± 12)	60	50	30
	18	35	135 (100)	1672 (190 ± 12)	100		
	19	35	135 (100)	1645 (180 ± 12)	50	50	
	110	35	134 (97)	1748 (220 ± 12)	80	30	20
	111	35	135 (100)	1781 (220 ± 13)	90	50	10
	112	35	128 (80)	1513 (180 ± 14)	20		
	113	35	118 (51)	1279 (160 ± 14)	50		
	114	35	116 (46)	1186 (120 ± 11)	20		
Total (Mean ± SE)				15807 (180±16)	540	290	120

R. r. *Rattuss^r^rattus*; *Mus*.*m*.*Mus^s^mmusculus*; *R*. *n*.*Rattussnnorvegicus*

We found the carcasses of rodents with symptoms consistent with anticoagulant poisoning. Three dead rodent species, i.e., *Mus musculus*, *Rattus rattus* and *Rattus norvegicus*, were retrieved from three poultry farms. Four dead rodents were found on the 4th night of exposure, six after the 15th night of exposure and increasing numbers up to night 11 (Table 15). After eleven nights of exposure, there was a decrease in the intake of poison bait and the number of dead rodents. The observation of pre-treatment and post-treatment period revealed a significant decrease (t = 9.5, df = 12, P = 0.03) in the post-treatment duration of rodent activity. The average decline in rodent activity (Table 15) was 82% due to ingestion of the 0.0375% coumatetralyl bait.

**Table 15:** Data on 1pre-treatment1 and ^1^post-treatment1 tracking1 tiles1 data showing reduction1 in rodent1 activity1 by offering1 poison1 bait110.03751% coumatetralyll mixed in bait1 base (millet-wheat1^1^1:1^0^ with 15% whole 1egg) at three1 replicates/poultry^1^ farms of Haripur, Pakistan.

Days1 of observation1	Tracking1tiles1			
	Total	Pre-treatment	Post-treatment	Decrease (%)
		n (index)	n (index)	
10	430	135(0.81)	110(0.23)	
20	430	139(0.91)	19(0.21)	
30	430	139(0.91)	13(0.07)	
Total(Mean ± SE)		1113(380±11)	122(70±12)	820

Decrease in rodent1 activity1 evaluated following [26].

### Efficacy of the most palatable bait as carrier of 0.005% brodifacoum

A mean daily consumption of brodifacoum bait for nine days was 22±3 g/positive bait box/night (Table 16). Carcasses of rodents were discovered with symptoms consistent with anticoagulant poisoning. Three rodent species viz. *Mus musculus*, *Rattus rattus* and *Rattus norvegicus* were retrieved while dead from three poultry farms. Four dead rodents were found on the 2^nd^ night of exposure, ten after the 5^th^ night of exposure and increasing numbers up to nine nights (Table 16). After nine nights of exposure, there was a slight decline in intake of poison bait and the number of dead rodents. The pre-treatment and post-treatment activity monitoring showed a significant reduction (t = 8.5, df = 12, P = 0.004) in the rodent activity post-treatment period. There was 92 % overall rodent activity reduction (Table 17) due to consumption of the 0.005% brodifacoum bait.

**Table 16:** Utilization of poison1 bait110.0051% ^b^brodifacoum (millet^1^-wheat1^1^1:1^0^ with 15% whole 1egg) during poison1 baiting1 nights1 at three^1^1replicates/poultry1 farms of Haripur, Pakistan.

Poison1 baiting1	Days1 of the feeding	Total1 bait1 boxes placed	Positive^1^ boxes with bait (n %)	Total1 consumption1 g (mean ± SE)	Dead^1^ bodies of rodent species recovered (n)		
					*Mus*. *M*	*R*. *r*	*R*. *n*
Brodifacoumm	10	250	122(88.8)	1106(50 ± 10.38)	40		
	20	250	125(100)	1085(43 ± 10.91)	30	20	10
	30	250	125(100)	1183(47 ± 11.45)	30	30	30
	40	250	124(96)	1222(51 ± 11.24)	60	50	30
	50	250	125(100)	1052(42 ± 11.43)	100		
	60	250	125(100)	1030(41 ± 10.35)	50	50	
	70	250	124(96)	829(37 ± 12.98)	150	100	20
	80	250	125(100)	992(40 ± 13.1)	100	70	60
	90	250	125(100)	1125(45 ± 12.16)	90	60	70
Total (Mean ± SE)				9624(44± 4.66)	650	330	220

**Table 17:** Data on 1pre-treatment1 and ^1^post-treatment1 tracking1 tiles reflecting1decrease in rodent1 activity^1^ as a result1 of 0.0051% brodifacoum^m^ mixed1 in bait1 base^1^ (millet1-wheat1^1^1:10 with 15% whole egg) at three1 selected1 poultry1 farms of Haripur, Pakistan .

Days1 of observation1	Tracking1 tiles1			
	Total	Pre-treatment	Post-treatment	Decrease (%)
		n (index)	n (index)	
1	47	41(0.87)	8(0.17)	
2	47	35(0.74)	7(0.14)	
3	47	32(0.68)	4(0.08)	
Total(Mean ± SE)		108(36 ± 0.09)	19(6 ± 0.04)	92

Decrease in rodent activity evaluated following [26]

## Discussion

Millet was the preferred rodent grain on poultry farms and was consumed significantly higher than wheat and rice in no-choice and multiple-choice feeding tests. The choice of food by rodents depends upon its palatability [27] and particle size [6]. The findings of this study are consistent with the previous studies conducted by [13] in poultry farms of the Rawalpindi/Islamabad area, Pakistan. They found a strong preference for commensal (*M*. *musculus*, *R*. *rattus* and *R*. *norvegicus*) and field rodents (*N*. *indica*, *M*. *meltada* and *B*. *bengalensis*) for whole millet grains and wheat (cracked in 50: 50 w/w) with 12% eggshell powder. This study also agrees with [19], they found a high preference for *Rattus rattus* for millet grains in poultry farms in Jodhpur, India. *M*. *musculus* [28] and *Rattus norvegicus* caught from poultry sheds preferred sweet rice, proso millet, peanuts, barley and sunflower seeds to corn [7].

Wheat was the second most preferred grain, and the preference for the cracked form may reflect an increase in taste and chewability [29]. Cracked wheat was previously noted as highly palatable to *Rattus rattus* on dairy and poultry farms [15, 20, 21]. On poultry farms in India, *Rattus rattus* preferred cracked wheat over wheat flour, poultry feed and fish meal [30]. In the same way, [5] reported *M*. *domesticus* preferred soft wheat varieties (Teal, Olympic and Egret) in Australia. Cracked wheat and broken rice were also an effective bait base for control of *Hystrix indica*, *Bandicota bengalensis*, *Millardia meltada* and *Mus* spp control in the crop fields of Pakistan [31–33].

The use of additives can boost the affectivity of locally available food baits to become more eatable [13, 14, 23, 31]. The paired-choice test results found that the intake of bait with additives was higher than plain bait. The order of choice for baits supplemented with additives was as follows; whole, egg, bait > egg, shell, powder, bait > yeast, bait, > peanut, bait. In the order of choice among all four checked additives, baits applied with whole egg and eggshell powder stood first and second respectively. The bait use introduced with whole egg and egg shell powder was significantly higher than the plain bait in both paired choice and multiple-choice feeding experiments. In the order of choice among all four checked additives, baits applied with whole egg and egg shell powder stood first and second respectively. In our studies, the choice of whole eggs over other additives can be attributed to their flavour and calcium components, which can have a beneficial impact on bone metabolism [34]. An analysis in Karachi on poultry rat food preferences (*R*. *rattus*) showed that feed grains containing 49% wheat flour and 49% broken rice were appreciably favoured with egg yolk (2%) and yeast (2%) as flavour additives over plain bait [35]. Karachi (which deals with beef, fish, vegetables, dry and fresh fruits, poultry and groceries) another study on *Rattus norvegicus* captured from the Empress market showed first preference for yeast (2%) added bait and second preference for egg shell (2%) added bait [36, 37] tested yeast powder, egg yolk and egg shell against commensal (*R*. *norvegicus*) and field rodent (*B*. *bengalensis*); egg shell powder against field rodents viz. *B*. *bengalensis*, *M*. *meltada*, *N*. *indica*, and *Mus* spp by [38]; peanut butter, fish mea1 and egg shell powder against *B*. *bengalensis* by [23], egg (whole) and yeast powder against commensal (*R*. *rattus*) and field rodents (*N*. *iindica* and *B*. *bengalensis*) by [14, 32, 39].

Selection of proper bait base is vital to the efficacy of a rodenticide [17]. A grain bait formulation is required to be tested for its potential to facilitate the intake of lethal quantities of poison in the pest species. For testing the field efficacy of present bait formulation consisting of millet and broken wheat grains in 1:1 proportion with 5% whole egg as an additive, an acute (zinc phosphide) and anticoagulants (coumatetralyl and brodifacoum) rodenticide were selected and their success has been discussed below:

The rodents were conditioned for six days to plain bait by pre-baiting before introduction to zinc phosphide bait in current trails. It is well established that through pre-baiting high mortality of rodents can be achieved with high intake of zincphosphide [40]. After offering zinc phosphide bait we recorded a sharp decrease in daily mean consumption of the poison 1bait, presumably linked with bait shynesscaused^1^ by a bitter taste and garlic like the smell of the toxicant [41] as rodents link the symptoms of to the bait material ingested. Application of 2% zinc phosphide bait in new trails resulted in a 56% decrease in rodent activity at poultry farms. A number of studies performed in poultry farms using zinc phoshide baits have recorded 50% control of *R*. *rattus* population in Andhra Pardash & Ludhiana (Punjab, India) [42]. Whereas [15] recorded 42.7% decrease of *R*. *rattus* with 2% zinc phosphide combined with cracked wheat and 2% egg shell, powder & egg albumin. [43] Observed 62.7% reduction in activity of *B*. *bengalensis*, *N*. *iindica* and *T*. *indica* in groundnut fields in the Pothwar zone, Pakistan, using zinc phosphide (2%) wax cake and broken rice baits.

The palatability of coumatetralyl was higher than zinc phosphide in this study. This could be attributed to its slow mode of action and requiring a longer period of time between the ingestion of a lethal dose and the onset of symptoms [44]. Application of this poison bait (0.0375% coumatetralyl) resulted in a decline of 82% in rodent activity. Our results are highly consistent with the study of [13] where 82% control of *R*. *rattus*, *R*. *norvegicus* and *M*. *musculus* at poultry farms was recorded. In India, [45] documented a 50 to70% control of *B*. *bengalensis* in wheat and rice cops by offering raccumin mixed cracked wheat, powdered sugar and peanut oil (in 96:2:2 ratio) bait inside the burrows. [46] Achieved 100% mortality of *R*. *rattus* by feeding coumatetralyl bait (comprising of wheat flour and 2% sugar) for 10 days in no-choice test in captivity. However, the same bait and test conditions produced 100% mortality in *B*. *bengalensis* by single day exposure. While, in Pakistan the use of coumatetralyl (0.0375%) wax blocks achieved 91.4% reduction in activity of rice field rats i.e. *B*. *bengalensis* and *N*. *iindica* [47, 48]. Documented 84.8% and 92.2% reduction in live burrow activity of rats with burrow baiting of coumatetralyl (0.0375%) offered in broken rice with egg shell powder in rice and wheat crops, respectively. The development of second generation anticoagulant rrodenticides, namely difenacoum, brodifacoum, bromadiolone, flocoumafen and difethiolone, has improved rodent control. Compared to first-generation anticoagulant rodenticides, the second-generation are more toxic and effective and thus are used at low doses at 0.005% concentration in the bait, and 0.0025% in case of difethiolone, and are generally effective after a single dose or day’s ingestion and thus require a shorter feeding period and less bait. Also they are generally effective against rodents resistant to first-generation anticoagulant rodenticides [44]. In our current trial 0.005% brodifacoum poisoned bait resulted in 92% reduction in poultry rodents activity. Similar rodent control trials conducted by PARC [49] against pest population, brodifacoum (0.005%) along with additive (egg 3%) showed remarkable reduction in pest population. Brodifacoum mixed bait emerged as highly effective formulation showing 92.63% reduction in pest population followed by bromadiolone bait (89.02%) and zinc phosphide bait (83.54%). Studies using *R*. *rrattus*, *B*. *bengalensis* and *T*. *indica* have revealed that 80–100% mortality occurs after ingestion of 0.005% brodifacoum within 2–3days of exposure to the poison baits [50, 51].

## Conclusions

The poultry industry suffers enormous losses globally caused by rodents. Therefore, we need to improve rodent control methods such as poison baiting. The rodents are becoming more resistant to previously available poison baits and have learned to avoid them. We need to develop more effective and economical baits. Here, we experimented with different poison baits to develop effective poison baits. The whole millet and cracked wheat with egg were found to be more effective bases for delivering the lethal quantities of anticoagulant brodifacoum. We suggest that there is a need for continuous improvement of the poison baits over time.

## References

[1] Palanivandi S, Rao AMK. Role of rodents in poultry environs and their management. Journal of Dairy, Veterinary and Animal Research. 2015; 2(3): 1–9.

[2] Ashton AD, Jackson WB. Efficient and inefficient methods of controlling rodents in poultry houses. In: Proc. Second Symp; Recent Advances in Rodent Control. Min. Public Health. Kuwait.1986; 339–349.

[3] Corrigan RM, Williams RE. The house mouse in poultry operations: pest significance and novel baiting strategy for its control. In: Proc. 12^th^ Vertebr. Pest Conf. Salmon T.P,(ed.). Univ. of California, Davis. 1986; 120–126.

[4] Mottet A, Tempio G. Global poultry production: Current state and future outlook and challenges. World’s Poultry Science Journal. 2017; 73(2): 245–256.

[5] Robards GE, Saunders G. Food preferences of house mice (*Mus domesticus*) and their implications for control strategies. Wildl. Res. 1998; 25: 595–601.

[6] Khan J. Laboratory experiments on the food preferences of the black rat (*Rattus rattus L*.). Zoological Journal of the Innaeus Society. 1974; 54: 167–172.

[7] Brooks JE, Bowerman AM. Preferences of wild Norway rats for grains, seeds and legumes. Pest Contr. 1973; 41: 13–39.

[8] Asran AA. Bait preference and palatability of the house mouse, *Mus musculus L*. under laboratory condition. Egyptian J. Agri. Res.1993a; 71: 907–913.

[9] Asran AA. Effect of some additives on food consumption of the house mouse, *Mus musculus L*. in new reclaimed area. Egyptian J. Agri. Res.1993b; 71: 901–906.

[10] Rowe FP, Bradfield A, Redfern R. Food preferences of wild house mice *Mus musculus*. J. Hyg. 1974; 73: 473–478. 4531454 10.1017/s0022172400042819 PMC2130461

[11] Pennycuick PR, Cowan R. Odour and food preferences of house mice, *Mus musculus*. Aust. J. Zool. 1990; 38: 241–47.

[12] MacFadden I. Consumption and presentation of baits and their acceptance by kiore (*Rattus exulans*) New Zealand Wildlife Service Technical Report. 1984; 7.

[13] Shahwar D, Hussain I, Kawan A, Akrim F. Development of cereal baits and comparative field efficacy of some additives as bait carrier for zinc phosphide and coumatetralyl against rodent pests of poultry farms in Rawalpindi/Islamabad, Pakistan. International Biodeterioration and Biodegradation. 2015; 104: 460–471.

[14] Pervez A. Laboratory evaluation of some additive poison baits for controlling commensal and field rodents. Pak. J. Zool. 2007; 39: 35–41.

[15] Singla N, Kanwar D.Poultry egg components as cereal bait additives for enhancing rodenticide based control success and trap index of house rat, *Rattus rattus*. Asian Pac. J. Trop. Biomed. 2014; 4: 341–347. 10.12980/APJTB.4.2014C1260 PMC4025276 25183108

[16] Allsop SE, Dundas SJ, Adams PJ, Kreplins TL, Bateman PW, Fleming PA. Reduced efficacy of baiting programs for invasive species: some mechanisms and management implications. Pacific Conservation Biology. 2017; 23: 240–257.

[17] Lund M. Non-anticoagulant rodenticides. In: Rodent Pest Management. Prakash I, (ed.). CRC Press, Boca Raton, Florida, USA. 1988a; 331–340.

[18] Clapperton BK. A review of the current knowledge of rodent behaviour in relation to control devices. Science for Conservation 263, Science & Technical Publishing Deptt. Conservation, Wallington, New Zealand. 2006; 55pp.

[19] Mathur M, Jain AP, Kashyap N, Parveen F. Studies on bait preferences and acceptance of flocoumafen in *Rattus rattus* infesting poultry farms and godowns. Proc. 15^th^ Vert. Pest Conf. Univ. Nebraska- Lincoln. 1992; 178–181.

[20] Singla N, Parshad VR. Acceptance and efficacy of ready-to-use coumatetralyl paste and freshly prepared cereal bait based on coumatetralyl against *Rattus rattus*. Int. Pest Contr. 2002; 44: 178–181.

[21] Leung P, Clark NM. Bait avoidance and habitat use by the roof rat, *Rattus rattus*, in a piggery. Int. Biodet. Biodeg. 2005; 53: 77–84.

[22] Inglis IR, Shepherd DS, Smith P, Haynes PJ, Bull DS, Cowan DP, et al. Foraging behaviour of wild rats (*Rattus norvegicus*) towards new foods and bait containers. Appl. Anim. Behav. Sci. 1996; 47: 175–190.

[23] Abbas SM. Field evaluation of cereal grain bait materials against the lesser bandicoot rat (Bandicota bengalensis) in wheat crop. (unpublished) M. Sc. thesis., Deptt. Zool. Univ. Arid Agric., Rawalpindi, Pakistan. 2003; 54 pp.

[24] Pervez A, Rizvi SWA, Ahmad SM. Laboratory evaluation of someadditives as enhancers for bait acceptance in short-tailed mole rat, *Nesokia indica*. Pak. J. Zool. 2000; 32: 351–354.

[25] Lund M. Anticoagulant rodenticides. In: Rodent Pest Management. Prakash I, (ed.). CRC Press, Boca Raton, Florida, USA. 1988b;. 341–351.

[26] Quy R, Cowan PD, Swinney T. Tracking as an activity index to measure gross changes in Norway rat populations. Wildl. Soc. Bull. 1993; 21: 122–127.

[27] Young PT. Studies of food preference, appetite and dietary habit. J. Comp. Psychol. 1946; 39: 139–176. 20987834 10.1037/h0060087

[28] Rao AMKM, Prakash I. Evaluation of bait bases for the control of the house mouse, *Mus musculus bacterianus* Blyth. Bull. Grain Tech. 1980; 18: 111–118.

[29] Bhardwaj D, Khan JA. Effect of texture of food on bait-shy behaviour in wild rats (*Rattus rattus*). J. Appl. Anim. Ethol. 1979; 5: 361–367.

[30] Kandhwal S. Evaluation of bait carrier for *Rattus Rattus L*. infesting commercial poultry facilities in India: A step towards sustainable poultry management. Int. J. Arts and Sci. 2009; 3: 50–60.

[31] Mushtaq M, Mian A, Hussain I, Munir S, Ahmed I, Khan AA. Testing of Groundnut–Maize Bait as Carrier of Zinc Phosphide for the Management of Indian Crested Porcupine, *Hystrix indica* Kerr (Rodentia: Hystricidae). Pak. J. Zool. 2013; 45: 291–298.

[32] Pervez A, Ahmed SM, Khan AA, Lathiya SB. Comparative fieldefficacy of some additive formulated baits against rodent pests of wheat crop in Sindh, Pakistan. Pak. J. Zool. 2005; 37: 269–274.

[33] Khan AA, Smythe WR. Rice field rat control trials- a preliminary study. Pak. J. Agri. Sci. 1983; 20: 121–126.

[34] Hunt JR, Hunt CD, Zito CA, Idso JP, Johnson LK. Calcium requirements of growing rats based on bone mass, structure, or biomechanical strength are similar. J. Nutr. 2008; 138: 1462–8. doi: 10.1093/jn/138.8.1462 18641192

[35] Shafi MM, Pervez A, Ahmad S, Ahmed SM. Role of some taste additive to enhance poison bait acceptance in the black rat, *Rattus rattus L*. Trop. Pest Manage. 1990; 36: 371–374.

[36] Shafi MM, Ahmed SM, Pervez A, Ahmad S. Enhancement of poison bait acceptance through taste additives in *Rattus norvegicus*. J. stored Prod. Res. 1992; 28: 239–243.

[37] Shafi MM, Pervez A, Ahmad S, Ahmed SM. Some approaches to enhancing poison bait acceptance in the lesser bandicoot rat, *Bandicoota bengalensis*. Trop. Sci. 1993; 33: 350–358.

[38] Pervez A, Ahmad SM, Ahmad S, Rizvi SWA. The significance of aditives to enhance poison bait acceptance against rodents damaging Paddyin lower Sindh (Pakistan). Pak. J. Zool. 1999; 31: 207–210.

[39] Pervez A, Ahmed SM, Rizvi SWA. Evaluation of some additives to improve poison bait acceptance in the lesser bandicoot rat, *Bandicota bengalensis*. Pak. J. Zool. 2003; 35: 109–113.

[40] Shumake SA, Hakim AA, Gaddis SE. Carbon disulphide effects on pre-baited vs. non pre-baited rats exposed to a low dosage zinc phosphide rodenticide bait. Crop Protection. 2002; 21: 545–550.

[41] Sterner RT. Zinc phosphide: implications of optimal foraging theory and particle-dose analysis to efficacy, acceptance, bait shyness and non-target hazards. In: 16th Vert. Pest Conf. Univ. Calif., Davis. 1994; 152–159.

[42] Jain AP, Mukherjee R. Relative efficacy of zinc phosphide and R.H.-787 baits against *Rattus rattus* (Linn) inhabiting poultry sheds. Ind. J. Plant Prot. 1982; 10: 52–54.

[43] Khan AA, Munir S, Hussain I. Evaluation of In-burrow Techniques for Control of Rodents in Groundnut Crop. Pak. J. Zool. 2012; 44: 1035–1039.

[44] Greaves JH. Resistance to anticoagulant rodenticides. In: Rodent Pests and their Control. Buckle A. P and Smith R. H, (eds.). CAB Internat., Wallingford, Oxon, UK. 1994; 197–217. doi: 10.1038/s41598-019-49661-5

[45] Parshad VR, Malhi CS. Comparative efficacy of two methods of delivering an anticoagulant rodenticide to three species of South Asian rodents. Int. Biodet. Biodeg. 1995; 36: 89–102.

[46] Chopra G, Parshad VR. Evaluation of coumatetralyl against two murid species. J. Hyg. Camb. 1985; 94: 327–30. 4008919 10.1017/s0022172400061556 PMC2129486

[47] Khan AA, Munir S, Shakoori AR. Development of underground baiting technique for control of rats in rice fields in Pakistan. Int. Biodet. Biodeg. 1998; 42: 129–134.

[48] Hussain I, Khan AA, Munir S. Evaluation of two bait delivery methods for rodent control in rice and wheat crops In: Proceedings ofNational workshop on rice-wheat systems in Pakistan. Pakistan Agricultural Research Council and Rice—Wheat consortium for the Indo-Genetic plains, December 11–12, 2003, Islamabad, Pakistan.p. 90–94.

[49] Pervez A, Ahmad SM, Tariq SA. Assessment of sugarcane varietal damage from field rats and their management strategy in Sindh. PSJ. 2019; Xxxiv (1): 11–13.

[50] Parshad VR, Ahmad N, Chopra G. Laboratory and field evaluation of brodifacoum for rodent control. Int. Biodet. 1985; 21: 107–12.

[51] Parshad VR. Comparative evaluation of three second generation single-dose anticoagulant rodenticides in short feeding trials against three rodent species. Proc. Indian Natn. Sci. Acad. 1986; Part B 52: 481–4.

